# Underwater Endoscopic Submucosal Dissection under General Anesthesia for Superficial Esophageal Carcinoma

**DOI:** 10.1055/a-2905-0019

**Published:** 2026-07-16

**Authors:** Yusuke Takahashi, Kotaro Shibagaki, Satoshi Kotani, Shinsuke Suemitsu, Shinsaku Tanaka, Masahito Aimi, Koichiro Furuta, Hideaki Mori, Norihisa Ishimura, Tetsuro Nikai, Shunji Ishihara

**Affiliations:** 1 Department of Gastroenterology 175764 Shimane University Faculty of MedicineIzumoShimane PrefectureJapan; 2 Department of Endoscopy 175764 Shimane University HospitalIzumoShimane PrefectureJapan; 3 Department of Gastroenterology 26415 Tottori City HospitalTottoriTottori PrefectureJapan; 4 Department of Gastroenterology 26817 National Hospital Organisation Hamada Medical CenterHamadaShimane PrefectureJapan; 5 Department of Anesthesiology 175764 Shimane University Faculty of MedicineIzumoShimane PrefectureJapan

**Keywords:** general anesthesia, muscularis propria exposure, post-ESD stenosis, superficial esophageal neoplasia

## Abstract

**Background and study aims** Conventional endoscopic submucosal dissection (C-ESD) for superficial esophageal carcinomas (SECs) can cause muscularis propria injury and post-ESD stenosis. Underwater ESD (U-ESD) facilitates muscle-sparing dissection but has been limited in the esophagus by aspiration concerns. This retrospective multicenter study evaluated the safety and effectiveness of esophageal U-ESD under general anesthesia.

**Patients and methods** U-ESD under general anesthesia was selected for lesions ≥30 mm, ≥half circumferential involvement, post-treatment scarring, or aspiration/paradoxical agitation risk; other lesions underwent C-ESD. Primary outcome was muscularis propria exposure (MPE). Secondary outcomes were post-ESD stenosis, resection speed, en bloc/R0 resection, and adverse events. Given the predefined treatment allocation, outcomes were compared between groups on an exploratory analysis. Post-ESD stenosis was further analyzed according to MPE in the U-ESD group.

**Results** Overall, 176 lesions were analyzed (U-ESD, 103; C-ESD, 73). The U-ESD group had larger lesions (45.2 vs. 22.1 mm) and more frequent ≥3/4 circumferential resection (70.8% vs. 6.8%). En bloc resection was achieved in all lesions; R0 resection rates were 96% and 94%, respectively. Resection speed was higher (15.2 vs. 7.6 mm ^2^ /min, *p* < 0.001) and MPE was less frequent (10.6% vs. 47.9%, *p* < 0.001) in the U-ESD group. Post-ESD stenosis was more frequent in the U-ESD group (11.6% vs. 1.3%, *p* = 0.015). Adverse events occurred only in the C-ESD group (4.1%). In the U-ESD group, post-ESD stenosis was more frequent with MPE than without MPE (45.4% vs. 7.6%, *p* = 0.001).

**Conclusion** Esophageal U-ESD under general anesthesia appeared safe and efficient for large, technically demanding SECs, achieving both infrequent MPE and high resection speed.

## Introduction

Advances in endoscopic submucosal dissection (ESD) provide a minimally invasive, organ-preserving alternative to surgery for superficial esophageal carcinomas (SECs). The 2022 Japan Esophageal Society guidelines extended ESD eligibility to lesions with endoscopically suspected slight submucosal invasion, as a subset of these lesions are histologically confined to the lamina propria. 1 Consequently, ESD is increasingly applied to large and technically demanding SECs.

Conventional ESD (C-ESD) is typically performed under deep sedation. However, paradoxical agitation, often observed in SEC patients with a history of heavy alcohol consumption, 2 3 may cause patient movement during the procedure, resulting in muscularis propria exposure (MPE), an endoscopically evident muscular injury that may precede perforation and has been associated with post-ESD stenosis. 4 5 Underwater ESD (U-ESD) has been reported to mitigate this risk of muscularis propria injury by increasing submucosal thickness through buoyancy and hydrostatic pressure, with favorable outcomes in gastric, duodenal, and colorectal ESD, 6 7 8 9 but its application to esophageal lesions has been limited by concerns regarding aspiration. General anesthesia with endotracheal intubation can overcome this limitation, enabling safe performance of esophageal U-ESD, while also providing a stable operative field and improving endoscopic control. 10 11 12

This retrospective multicenter study evaluated the safety and effectiveness of esophageal U-ESD under general anesthesia for SECs, using MPE as the primary endpoint to assess clinically relevant muscular injury, with an exploratory comparison with C-ESD.

## Patients and Methods

### Participants

This multicenter retrospective observational study included consecutive patients with SECs who underwent ESD at Shimane University Hospital, Tottori City Hospital, or Hamada Medical Center between April 2021 and June 2025. Cases were excluded if they met any of the following criteria: lesion size <10 mm, additional surgical treatment, use of a combined knife-and-snare technique, unavailable or inadequate post-ESD ulcer-bed images and/or procedure videos precluding assessment of muscularis propria exposure, or deviation from the predefined post-ESD stenosis prevention and follow-up protocol.

The protocol was approved by the institutional review boards of all participating institutions, and informed consent was obtained using an opt-out approach. The study was conducted according to the Declaration of Helsinki and was reported following the Strengthening of the Reporting of Observational Studies in Epidemiology (STROBE) statement. 13

### Management of Deep Sedation and General Anesthesia

C-ESD was performed under deep sedation using midazolam (4–10 mg), pethidine hydrochloride (35–70 mg), and dexmedetomidine (0.4 μg/kg/h). U-ESD was performed under general anesthesia administered and continuously monitored by anesthesiologists, using intravenous agents including propofol, remifentanil, and rocuronium in accordance with institutional protocols. General anesthesia was selected according to predefined clinical criteria when at least one of the following conditions was present: lesion ≥30 mm, circumferential involvement of more than half of the esophageal lumen, lesion located in the cervical esophagus, proximity to post-treatment scarring, or a high risk of aspiration or paradoxical agitation.

### ESD Procedures

**Fig. 1:**
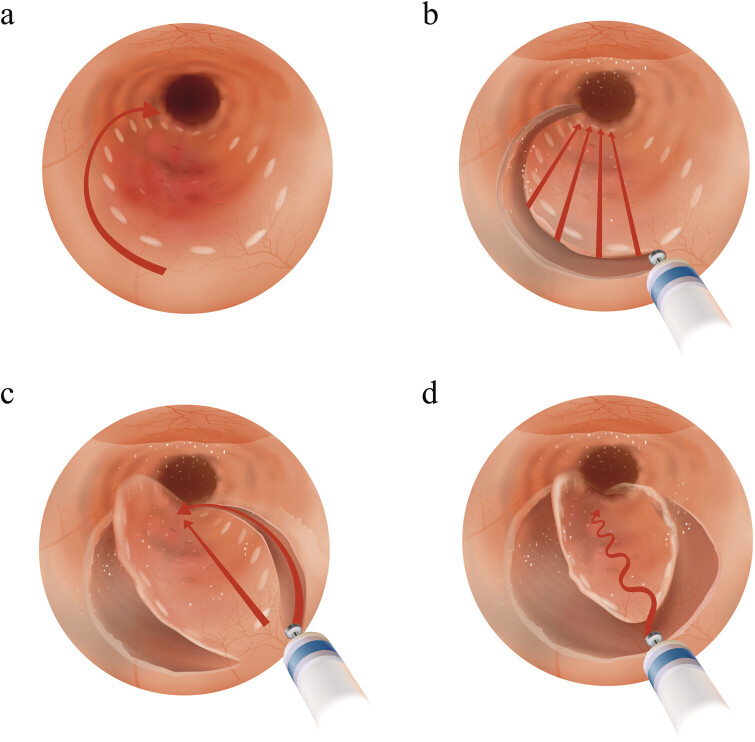
C-shaped incision technique for esophageal underwater ESD. ( **a** ) A C-shaped mucosal incision covering more than half of the circumference is made from the oral to the anal side. ( **b** ) Submucosal dissection is performed within the incision along the exposed submucosal layer, proceeding from oral to anal. ( **c** ) After completing dissection within the C-shaped area, the contralateral mucosa is incised while leaving a small oral mucosal bridge, and the remaining submucosa is dissected longitudinally. ( **d** ) After completing the circumferential incision, the remaining submucosal layer is dissected; clip-with-line traction is often applied as needed.

ESD was performed using the C-shaped incision method in all C-ESD and U-ESD cases, as schematically illustrated in Fig. 1 . A 1:1 mixture of sodium alginate and glycerol containing epinephrine (0.0125 mg/mL) was injected into the submucosa. The initial mucosal incision, covering more than half of the circumference, was made from the oral to the anal side, followed by submucosal dissection. Dissection was carefully controlled to preserve a thin submucosal layer above the muscularis propria. When esophageal glands were identified, dissection was performed beneath them to account for potential ductal involvement. 14 15 16 The opposite side was then incised, and the remaining submucosa was dissected longitudinally from the oral to the anal side. The thread-traction method was applied as needed. 17 Procedures were performed by expert endoscopists certified by the Japan Gastroenterological Endoscopy Society, each with experience in ≥50 esophageal ESD cases.

### Underwater Technique



**Video 1** Underwater endoscopic submucosal dissection (U-ESD) for an entirely circumferential superficial esophageal squamous cell carcinoma measuring 10 cm in longitudinal extent. Saline immersion increased submucosal thickness through buoyancy and hydrostatic pressure and enhanced visualization of esophageal glands, allowing stable deep-layer dissection beneath the glands while preserving the submucosal layer on the resection bed. En bloc curative resection was achieved without muscularis propria injury, followed by triamcinolone acetonide filling. Follow-up endoscopy at 6 months revealed re-epithelialization without significant stenosis.


**Fig. 2:**
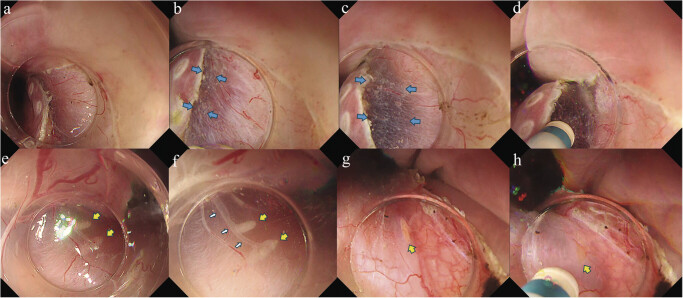
Underwater technique in the esophagus. ( **a)** Under air insufflation, submucosal dissection at the mucosal incision edge is challenging because of the limited working space. ( **b** ) Under saline immersion, buoyancy-assisted mucosal traction increases submucosal thickness, visualizing the submucosa as a bluish-white zone (blue arrows). ( **c** ) With additional water-jet infusion and the endoscope positioned parallel to the submucosal layer, the bluish-white submucosa further expands. ( **d** ) This facilitates precise dissection along the central plane of the submucosal layer while minimizing muscularis propria injury. ( **e** ) Under air insufflation, submucosal esophageal glands may be obscured by light reflection (yellow arrows). ( **f** ) Under saline immersion, glare is eliminated, enabling clear visualization of gland acini (yellow arrows) and ducts (white arrows). ( **g** ) Underwater identification of an esophageal gland in the submucosal layer (yellow arrow), with only a thin submucosal layer adjacent to the muscularis propria. ( **h** ) Additional water-jet infusion from the DualKnife thickens the submucosal layer beneath the gland, allowing safe removal while preserving the muscularis propria.

In U-ESD cases, the ESD procedure was performed with 0.9% saline immersion under general anesthesia ( Video 1 ). After air evacuation, saline was infused using an endoscopic water-jet to establish an underwater environment, with simultaneous submucosal injection and buoyancy-assisted mucosal traction. With the endoscope positioned parallel to the thickened submucosal layer, the submucosa appears bluish-white ( Fig. 2 , upper panels), allowing precise dissection along its central plane and minimizing muscularis propria injury. Underwater visualization enhances identification of submucosal esophageal glands that are often obscured by light reflection under air insufflation, while saline infusion elevates these glands, allowing dissection beneath them while preserving a thin submucosal layer ( Fig. 2 , lower panels). 18

### TA-filling Method for Stenosis Prevention

For lesions with a resection circumference of ≥ three-quarters, a triamcinolone acetonide (TA)-filling method, as previously described by our group, was performed according to a predefined protocol to prevent post-ESD stenosis. 19 In addition, TA filling was performed at the operator’s discretion for lesions with near-three-quarters circumferential resection when additional risk factors for post-ESD stenosis were considered present. Briefly, under sedation, the esophagogastric lumen was fully deaerated, and 4 mL of a TA–saline solution (80 mg) was slowly infused via the endoscope channel, followed by an additional 16–18 mL of saline to fill the esophagus with the TA solution. The patient was maintained in the left lateral position for 7 min to ensure adequate contact of the TA solution with the resection bed. This procedure was performed on the day of ESD and again 1 week later.

### Follow-up and Management

Follow-up endoscopy was performed every two weeks after the second TA filling until complete re-epithelialization without significant stenosis was confirmed. Patients without luminal narrowing underwent observation alone. Luminal narrowing allowing endoscope passage was treated with additional TA filling. Stenosis preventing endoscope passage was treated by endoscopic balloon dilation, followed by TA filling or local TA injection according to the extent of the residual non-epithelialized area.

### Endoscopic Equipment and Electrosurgical Settings

Both C-ESD and U-ESD were performed using a single-channel upper endoscope (GIF-Q260J or GIF-H290T; Olympus Medical Systems, Tokyo, Japan) equipped with a transparent distal attachment hood (ST Hood; Fujifilm, Tokyo, Japan ) , electrosurgical generators (VIO 300 D or VIO 3; ERBE Elektromedizin, Tübingen, Germany), and knives (DualKnife J and/or HookKnife J; Olympus). Mucosal incision and submucosal dissection were performed in Endocut I mode (VIO 300 D: effect 2; VIO 3: effect 1; duration 2 and interval 2 for both systems) to minimize thermal injury to the muscularis propria. Vascular precoagulation and hemostasis were achieved using spray coagulation (VIO 300 D: effect 2, 60 W; VIO 3: effect 4.7) or soft coagulation (VIO 300 D: effect 4, 50 W; VIO 3: effect 5).

### Clinical Outcomes and Definitions

**Fig. 3:**
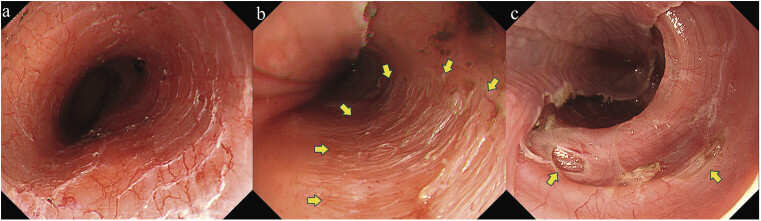
Representative endoscopic images of muscularis propria exposure. ( **a** ) No muscularis propria exposure after entirely circumferential ESD. ( **b** ) Muscularis propria exposure (≥5 mm), confined to the inner circular muscle layer. ( **c** ) Muscularis propria exposure involving multiple small sites.

The primary outcome was MPE, defined as visible exposure of the muscularis propria of ≥5 mm in length, corresponding to at least twice the tip diameter of the DualKnife J (2.7 mm), or exposure involving multiple sites 5 ( Fig. 3 ). In both groups, the post-ESD ulcer base was assessed after completion of ESD under air insufflation. MPE and the depth of muscular injury were determined by consensus of two expert endoscopists. For each lesion, all post-ESD ulcer-bed images were extracted and reviewed; when image quality or coverage was inadequate to determine the presence of MPE or the depth of muscular injury, the corresponding procedure videos were additionally reviewed to finalize the assessment. The assessors were blinded to subsequent post-ESD stenosis.

Secondary outcomes included post-ESD stenosis, the number of dilation sessions, resection speed (mm ^2^ /min), en bloc and R0 resection rates, and adverse events (e.g., perforation, bleeding, and aspiration pneumonia). Post-ESD stenosis was defined as failure to allow passage of a standard endoscope with an outer diameter of 9.8–9.9 mm, even with a pushing operation. Resection speed was calculated as the estimated specimen area (long axis/2 × short axis/2 × π) divided by the procedure time, defined as the interval from initial mucosal incision to completion of lesion removal.

### Statistical Analysis

As treatment allocation was not randomized and U-ESD under general anesthesia was selected according to predefined clinical criteria, baseline characteristics and clinical outcomes were compared between groups on an exploratory analysis. Within the U-ESD group, post-ESD stenosis and the number of dilation sessions were compared according to MPE status. Continuous variables were expressed as mean ± standard deviation or median (range), depending on data distribution. Continuous variables were compared using Student’s *t* test, Welch’s *t* test, or the Mann–Whitney *U* test, as appropriate. Categorical variables were compared using the chi-square test or Fisher’s exact test. All tests were two-sided, and *p* values < 0.05 were considered statistically significant.

## Results

**Fig. 4:**
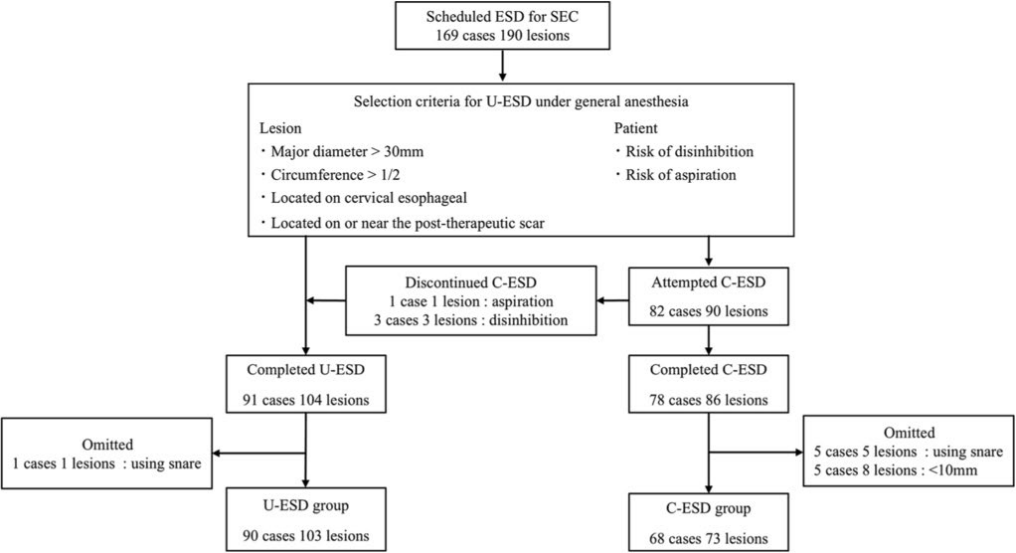
Study flow diagram of patient enrolment and allocation to the U-ESD and C-ESD groups.

A total of 169 patients with 190 SEC lesions underwent ESD. C-ESD was initially attempted in 82 patients (90 lesions), but the procedure was aborted in 4 patients (4 lesions) and rescheduled as U-ESD under general anesthesia because of paradoxical agitation ( *n* = 3) or aspiration ( *n* = 1); these cases were analyzed in the U-ESD group. Patients treated with a combined knife-and-snare technique (6 patients, 6 lesions) and those with lesions <10 mm (5 patients, 8 lesions) were excluded. No lesions were excluded because of additional surgical treatment. Consequently, 158 patients with 176 lesions were finally enrolled, including 90 patients with 103 lesions in the U-ESD group and 68 patients with 73 lesions in the C-ESD group ( Fig. 4 ). All included lesions had sufficient post-ESD ulcer-bed images and/or procedure videos to assess muscularis propria exposure, and the predefined post-ESD stenosis prevention and follow-up protocol was adhered to.

### Clinicopathological Characteristics

Clinicopathological characteristics are summarized in Table 1 . Sex, age, tumor location, histological type, and use of the thread-traction method did not differ between the U-ESD and C-ESD groups. The U-ESD group had larger lesions (45.2 vs. 22.1 mm, *p* < 0.001) and more frequent muscularis mucosae or deeper invasion (32.0% vs. 17.8%, *p* = 0.034) than the C-ESD group. Thread traction was used in 61 U-ESD lesions (59.2%) and 36 C-ESD lesions (49.3%), without a significant difference between groups ( *p* = 0.283).

**Table 1:** Clinicopathological characteristics.

	U-ESD (90 cases, 103 lesions)	C-ESD (68 cases, 73 lesions)	*p* -Value
Sex, male/female ( *n* )	87/3	61/7	0.123
Age (mean ± SD, year)	71.0 ± 10.1	72.6 ± 8.9	0.235
Location, *n* (%)			0.70
*Upper esophagus*	24 (23.3%)	19 (26.0%)	
*Middle esophagus*	57 (55.3%)	36 (49.3%)	
*Lower esophagus*	22 (21.4%)	18 (24.7%)	
Lesion size (mean ± SD, mm)	45.2 ± 19.5	22.1 ± 7.8	<0.001
Histological type, *n* (%)			0.379
*Squamous cell carcinoma*	99 (96.1%)	68 (93.2%)	
*Adenocarcinoma*	4 (3.9%)	5 (6.8%)	
Invasion depth, *n* (%)			0.034
*Epithelium or Lamina propria mucosae*	70 (68.0%)	60 (82.2%)	
*Muscularis mucosae or Submucosa*	33 (32.0%)	13 (17.8%)	
Use of the thread-traction method, *n* (%)	61 (59.2%)	36 (49.3%)	0.283

U-ESD, Underwater endoscopic submucosal dissection; C-ESD, Conventional endoscopic submucosal dissection; SD, standard deviation.

### Clinical Outcomes in the U-ESD and C-ESD Groups

Clinical outcomes are shown in Table 2 . En bloc resection was achieved in all cases, with comparably high R0 resection rates (96% in the U-ESD group vs. 94% in the C-ESD group, *p* = 0.617). The resection size and resection area were larger in the U-ESD group than in the C-ESD group (52.0 vs. 30.0 mm and 1636 vs. 560 mm ^2^ , respectively; both *p* < 0.001). Sub-circumferential or circumferential resection was more frequent in the U-ESD group (70.8% vs. 6.8%, *p* < 0.001).

**Table 2:** Outcomes of underwater vs. conventional esophageal ESD.

	U-ESD ( *n* = 103)	C-ESD ( *n* = 73)	*p* -Value
En bloc resection, *n* (%)	103 (100%)	73 (100%)	1.000
R0 resection, *n* (%)	99 (96%)	69 (94%)	0.617
Resection size (mean ± SD, mm)	52.0 ± 20.7	30.0 ± 8.5	<0.001
Resection circumference, *n* (%)			<0.001
*<3/4 of the circumference*	30 (29.1%)	68 (93.2%)	
*≥3/4 of the circumference, sub-circumferential*	62 (60.2%)	5 (6.8%)	
*Entirely circumferential*	11 (10.7%)	0	
Resection area (mean ± SD, mm ^2^ )	1636 ± 1283	560 ± 323	<0.001
Procedure time (mean ± SD, min)	102 ± 52	76 ± 29	0.002
Resection speed (mean ± SD, mm ^2^ /min)	15.2 ± 6.2	7.6 ± 3.2	<0.001
Muscularis propria exposure, *n* (%)	11 (10.6%)	35 (47.9%)	<0.001
Steroid treatment after ESD, *n* (%)	94 (91.3%)	12 (16.4%)	<0.001
Post-ESD stenosis, *n* (%)	12 (11.6%)	1 (1.3%)	0.015
Number of dilation sessions, median (range)	1 (1–2)	1 (1)	0.569
Adverse events, *n* (%)	0 (0%)	3 (4.1%)	0.092

U-ESD, Underwater endoscopic submucosal dissection; C-ESD, Conventional endoscopic submucosal dissection; SD, standard deviation.

Procedure time was longer in the U-ESD group (102 ± 52 vs. 76 ± 29 min, *p* = 0.002), but resection speed was about twice as fast (15.2 vs. 7.6 mm ^2^ /min, *p* < 0.001). MPE at the resection bed was observed significantly less frequently in the U-ESD group (10.6% vs. 47.9%, *p* < 0.001); among cases with MPE, involvement was confined to the inner circular muscle layer in most cases (U-ESD, 10/11; C-ESD, 33/35), with extension to the outer longitudinal layer in only three cases overall (1 in U-ESD and 2 in C-ESD). The incidence of post-ESD stenosis was higher in the U-ESD group (11.6% vs. 1.3%, *p* = 0.015); all cases of post-ESD stenosis occurred in lesions with ≥ three-quarters circumferential involvement. Adverse events occurred only in the C-ESD group (0% vs. 4.1%, *p* = 0.092): aspiration pneumonia, perforation, and delayed bleeding (one case each). No complications related to general anesthesia occurred in the U-ESD group.

### Outcomes by MPE in the U-ESD Group

Table 3 summarizes clinicopathological features and outcomes according to MPE status in the U-ESD group. Lesion location and invasion depth were comparable between the MPE and non-MPE groups. Lesion size (49.8 vs. 44.6 mm, *p* = 0.517) and resection size (57.4 vs. 48.4 mm, *p* = 0.350) were numerically larger in the MPE group, and sub-circumferential or circumferential resection was numerically less frequent (63.6% vs. 71.7%, *p* = 0.512). TA filling was performed in all MPE cases and most non-MPE cases (100% vs. 90.2%, *p* = 0.278). Post-ESD stenosis was more frequent in the MPE group (45.4% vs. 7.6%, *p* = 0.001), whereas the median number of dilation sessions did not differ (1 [range, 1–2] vs. 1 [range, 1–2], *p* = 0.735).

**Table 3:** Outcomes by muscularis propria exposure in underwater ESD.

	Muscularis propria exposure		*p* -Value
	Present ( *n* = 11)	Absent ( *n* = 92)	
Location, *n* (%)			0.778
*Upper esophagus*	3 (27.3%)	22 (23.9%)	
*Middle esophagus*	5 (45.4%)	52 (56.5%)	
*Lower esophagus*	3 (27.3%)	18 (19.6%)	
Lesion size (mean ± SD, mm)	49.8 ± 24.9	44.6 ± 18.8	0.517
Invasion depth, *n* (%)			0.745
*Epithelium or Lamina propria mucosae*	7 (63.6%)	63 (68.5%)	
*Muscularis mucosae or Submucosa*	4 (36.4%)	29 (31.5%)	
Resection size (mean ± SD, mm)	57.4 ± 26.7	48.4 ± 17.8	0.350
Resection circumference, *n* (%)			0.512
*< 3/4 of the circumference*	4 (36.4%)	26 (28.3%)	
*≥3/4 of the circumference, sub-circumferential*	5 (45.4%)	57 (61.9%)	
*Entirely circumferential*	2 (18.2%)	9 (9.8%)	
Resection speed (mean ± SD, mm ^2^ /min)	14.2 ± 7.6	15.6 ± 5.8	0.611
Steroid treatment after ESD, *n* (%)	11 (100%)	83 (90.2%)	0.278
Post-ESD stenosis, *n* (%)	5 (45.4%)	7 (7.6%)	0.001
Number of dilation sessions, median (range)	1 (1–2)	1 (1–2)	0.735

ESD, Endoscopic submucosal dissection; SD, standard deviation.

## Discussion

**Fig. 5:**
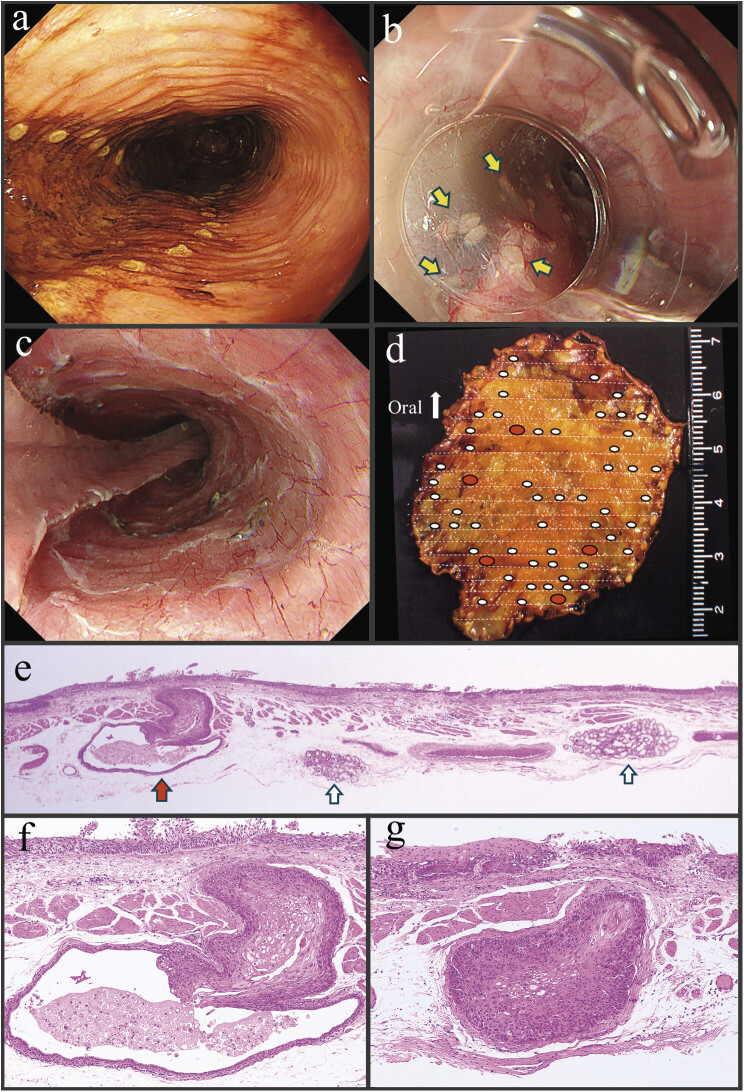
Ductal involvement of superficial esophageal squamous cell carcinoma in submucosal esophageal glands. ( **a** ) Superficial esophageal squamous cell carcinoma with ≥ three-quarters circumferential extension treated by ESD. ( **b** ) During submucosal dissection, numerous submucosal esophageal glands were observed (yellow arrows), and dissection was performed beneath the glands. ( **c** ) Sub-circumferential dissection was completed with a thin submucosal layer preserved over the muscularis propria. ( **d** ) Schematic mapping of submucosal esophageal glands in the resected specimen: glands are indicated by white circles and ductal involvement by red circles (five sites). ( **e** ) Low-power view shows extensive intraepithelial carcinoma with ductal involvement in a submucosal gland (red arrow) among multiple glands (white arrowheads). ( **f** ) High-power view demonstrates lateral intraductal extension through a duct penetrating the muscularis mucosae. ( **g** ) Another involved duct shows intraductal extension reaching the deep submucosa (maximum depth, 435 μm from the muscularis mucosae).

In the esophagus, U-ESD has distinct organ-specific implications beyond its technical advantages. Esophageal glands located in the submucosa are involved intraductally in 21.3–38.1% of superficial squamous cell carcinomas, 14 15 16 and incomplete gland removal may result in residual carcinoma. However, because these glands are often situated deep within the submucosal layer, complete removal increases the risk of muscularis propria injury. The water-jet effect of U-ESD promotes the formation of a thick submucosal layer beneath the glands, thereby mitigating electrocautery-related injury to the muscularis propria ( Fig. 5 ). Thread traction method was used on an as-needed basis in 59.2% of U-ESD lesions. Although water pressure and buoyancy may facilitate submucosal entry and provide intrinsic mucosal traction, mechanical traction remains a useful complementary maneuver when stable exposure is required during esophageal U-ESD.

For esophageal U-ESD, general anesthesia was selected to minimize aspiration risk. Previous studies have shown that general anesthesia is associated with faster resection speed, 10 higher R0 resection rates, and lower perforation rates 11 in esophageal ESD, likely owing to anesthesiologist-managed airway control and complete patient immobility, which allow endoscopists to focus on precise endoscopic manipulation. However, these reports evaluated CO₂-insufflated esophageal ESD for relatively small lesions (median size, 18–22 mm), with sub-circumferential or greater resections accounting for only 0–25.2% of cases. 10 11 12 Even in such cohorts, post-ESD stenosis occurred in 7.8%, 10 and adverse events—mainly mediastinal emphysema—were reported in up to 12.2%. 12 In contrast, in the present study, 70.9% of lesions treated with U-ESD under general anesthesia required sub-circumferential or circumferential resection; nevertheless, a high R0 resection rate of 96% was maintained, resection speed was approximately twofold higher than that with C-ESD, no adverse events were observed, and post-ESD stenosis was limited to 11.6%. These findings suggest that combining U-ESD with general anesthesia may offer complementary advantages even in large and technically demanding esophageal lesions. Given the required resources, this approach should be reserved for selected lesions or patients in whom airway control, procedural stability, and muscle-sparing dissection are expected to be clinically relevant.

Post-ESD stenosis is the most frequent complication after extensive esophageal ESD, particularly in cases with resection circumference of ≥ three-quarters of the esophageal lumen. 20 21 22 Without prophylactic intervention, stenosis rates are reported to be 61–82% after sub-circumferential resection 23 24 25 26 and 100% after circumferential resection. 27 Local TA injection and oral prednisolone are standard preventive approaches, whereas the TA filling method used in the present study represents a simpler alternative. These steroid-based treatments reduce stenosis rates to 5–45% after sub-circumferential resection, 23 24 25 28 29 30 but rates remain high at 27–71% after circumferential resection. 25 31 32 In recent years, several studies have consistently identified muscularis propria injury as an independent risk factor for stenosis after extensive esophageal ESD. 5 33 34 Geng et al. demonstrated that MPE of ≥5 mm or involving multiple sites was associated with post-ESD stenosis in a large cohort of esophageal ESD cases, 5 whereas Azuma et al. reported that, in circumferential ESD cases, deep muscular injury reaching the outer longitudinal muscle was an independent risk factor for refractory stenosis. 34 These clinical observations are supported by experimental data showing that deep thermal injury reaching the muscularis propria results in mural fibrosis and subsequent stenosis. 35 In the present study, MPE was observed in 47.9% of C-ESD cases; however, post-ESD stenosis occurred in only 1.3% of cases, reflecting the limited resection circumference in most cases. In contrast, most patients in the U-ESD group underwent sub-circumferential or circumferential resection; however, MPE occurred in only 10.6% of cases and was predominantly confined to the inner circular muscle layer, resulting in a low overall post-ESD stenosis rate of 11.6%. Post-ESD stenosis in the U-ESD group developed in 45.4% of lesions with MPE, compared with only 7.6% of those without MPE. Most cases required only a single dilation session, and no refractory stenosis was observed, possibly reflecting the limited depth of muscular injury in U-ESD.

The present study has several limitations. First, this was a retrospective multicenter study with nonrandomized treatment allocation, and U-ESD under general anesthesia was preferentially selected for larger and more technically demanding lesions. As multivariable adjustment for MPE was not performed, residual confounding may have affected the between-group comparison of MPE. Second, although assessors were blinded to subsequent post-ESD stenosis, they were not blinded to the treatment group during MPE assessment, leaving potential assessment bias. Third, histological assessment of resection depth was not performed, precluding between-group comparison. Fourth, the limited number of post-ESD stenosis events precluded reliable multivariable adjustment for factors associated with stenosis. Finally, because anesthesia methods differed between groups, the effects of the underwater technique and general anesthesia could not be evaluated separately. Therefore, between-group comparisons should be interpreted as exploratory.

In conclusion, esophageal U-ESD under general anesthesia appeared to be a safe and efficient therapeutic option for large and technically demanding SECs, achieving both infrequent MPE and high resection speed.

**Abbreviations**
C-ESD: Conventional endoscopic submucosal dissection
ESD: Endoscopic submucosal dissection
MPE: Muscularis propria exposure
SEC: Superficial esophageal carcinoma
TA: Triamcinolone acetonide
U-ESD: Underwater endoscopic submucosal dissection
